# Thyroid disorders and inflammatory bowel disease: an association present in adults but also in children and adolescents

**DOI:** 10.3389/fendo.2025.1425241

**Published:** 2025-02-04

**Authors:** Valeria Calcaterra, Francesca Penagini, Virginia Rossi, Luisa Abbattista, Alice Bianchi, Massimiliano Turzi, Lucia Cococcioni, Gianvincenzo Zuccotti

**Affiliations:** ^1^ Pediatric and Adolescent Unit, Department of Internal Medicine, University of Pavia, Pavia, Italy; ^2^ Pediatric Department, Buzzi Children’s Hospital, Milano, Italy; ^3^ Department of Biomedical and Clinical Science, University of Milan, Milano, Italy

**Keywords:** thyroid disorders, cancer, children, adolescents, inflammatory bowel disease, Crohn’s disease, ulcerative colitis

## Abstract

Inflammatory bowel diseases (IBD) represent chronic inflammatory multisystemic disorders that primarily involve the gastrointestinal tract. Patients with ulcerative colitis (UC) and Crohn’s disease (CD) exhibit a higher prevalence of thyroid disorders compared to the general population. The aim of this review is to summarize the literature on concomitant IBD and thyroid disorders, specifically autoimmune thyroid diseases such as Graves’ disease (GD) and Hashimoto’s thyroiditis (HT), as well as thyroid cancer, with a focus on children and adolescents. We provide an overview of the age-related differences between children and adults in the prevalence of this association. Literature shows that relatively few studies have been conducted on this subject in pediatric populations. The etiopathogenetic similarities between IBD and autoimmune thyroiditis are undeniable. Nevertheless, current data does not indicate a unanimous association between GD and HT and chronic IBD (both CD and UC). Although evidence suggests a potential association between IBD and thyroid cancer, particularly papillary thyroid cancer, the precise nature of this relationship varies across studies and is influenced by multiple factors. The limited information regarding the relationship between IBD and thyroid disorders in children highlights a significant knowledge gap. Since the thyroid plays a critical role in the pediatric population’s development, it is essential to promptly recognize and treat thyroid diseases. A thyroid function monitoring and future research exploring the genetic and immunologic connections are essential to enhance our understanding of the interrelation between IBD and thyroid disorders.

## 1 Introduction

The term inflammatory bowel disease (IBD) refers to two main disorders, characterized by a chronic relapsing inflammation of the digestive tract. IBD includes Crohn’s disease (CD) and ulcerative colitis (UC). Their prevalence amongst the pediatric population has recently increased, probably due to better diagnostic tools and the spread of risk factors, with more than 5 children affected every 10000 subjects (1). Though genetic background has a strong association with the development of the disease, other risk factors have been recognized. These include passive smoking during pregnancy and early life, infections, altered fibers and sugar intake and some medications exposure. A protective factor is breastfeeding (2). Clinical manifestations are protean, but they usually involve the digestive system with abdominal pain, diarrhea (sometimes bloody diarrhea) and perianal lesions. Other non-gastroenteric manifestations are growth failure, weight loss, delayed puberty, joint pain, low grade fever, eye lesions, oral ulcerations, and skin lesions. Additionally, an increased rate of thyroid disorders is reported in patients with UC and CD than the normal population (3).

Thyroid disorders, functionally classified into thyrotoxicosis and hypothyroidism, include a lot of different types of diseases, including autoimmune thyroiditis, congenital thyroid disease, amyloid goiter, and, rarely, neoplasms. Hypothyroidism is the commonest form; etiologically, acquired variants are the most prevalent ones, especially Hashimoto’s thyroiditis. On the other hand, as for hyperthyroidism, the most frequent cause is Graves-Basedow disease (1, 4). IBD and autoimmune thyroiditis seems to share several etiopathogenetic mechanism but it’s still unclear if IBD patients have greater risk to present these thyroid disorders.

Individuals with IBD also exhibit an elevated risk of developing both gastrointestinal and extraintestinal malignancies. Persistent inflammation is identified as a contributing factor to carcinogenesis (5, 6). While earlier studies have suggested a heightened risk of colorectal cancer among IBD patients relative to the general population, recent evidence indicates a declining trend. This decrease is attributed to improved surveillance strategies, implementation of colectomy in certain regions, and effective management of inflammation (6–8). Concurrently, there is a rising incidence of extraintestinal cancers (EICs) (9, 10), with Crohn’s disease patients demonstrating a particularly increased risk. This predominantly involves skin and liver-biliary cancers, especially in those with concurrent IBD and primary sclerosing cholangitis (11). Even though the data reported is limited, much rarer are thyroid neoplasms. Thyroid cancer seem to be more prevalent in patients with IBD than the control group, specifically CD (12). Not well defined is the association with IBD and thyroid cancer in pediatrics.

The aim of this report is to review and summarize the literature on IBD and thyroid diseases, specifically thyroid autoimmune disorders and thyroid cancer, with a focus on children and adolescents, to support the regular monitoring of thyroid function. Most reports on this topic focus on adulthood, and only a few studies provide an overview of findings related to the pediatric-adolescent age group in comparison to adults. Given the thyroid's critical role in the development of the pediatric population, thyroid diseases are highly significant and must be promptly recognized and treated (13). Keeping the discussion open in this field can help establish new monitoring guidelines for the pediatric-adolescent population and facilitate a smoother transition into adulthood.

## 2 Methods

We conducted a narrative review (14) to explore the association between IBD and thyroid disorders. A comprehensive literature search was carried out using the PubMed electronic database, focusing on studies published in the last 25 years in English and Spanish. Both pediatric (subjects aged <18 years) and adult (subjects aged >18 years) studies were included in the search, with a clear distinction made between these populations during the analysis. When pediatric data were limited, adult data were used to highlight that the lack of pediatric studies might be a gap. The types of publications examined included original research articles, systematic reviews, meta-analyses, longitudinal studies, randomized studies. Case reports or small case series, non-peer-reviewed articles, editorials, or letters were excluded. For the research, the following keywords were used (alone and/or combined): intestinal bowel diseases; ulcerative colitis; crohn’s disease; pediatric population; childhood; children; thyropathies; hyperthyroidism; hypothyroidism; thyroid neoplasms; thyroid malignancies; thyroid cancers. 

**Figure 1:**
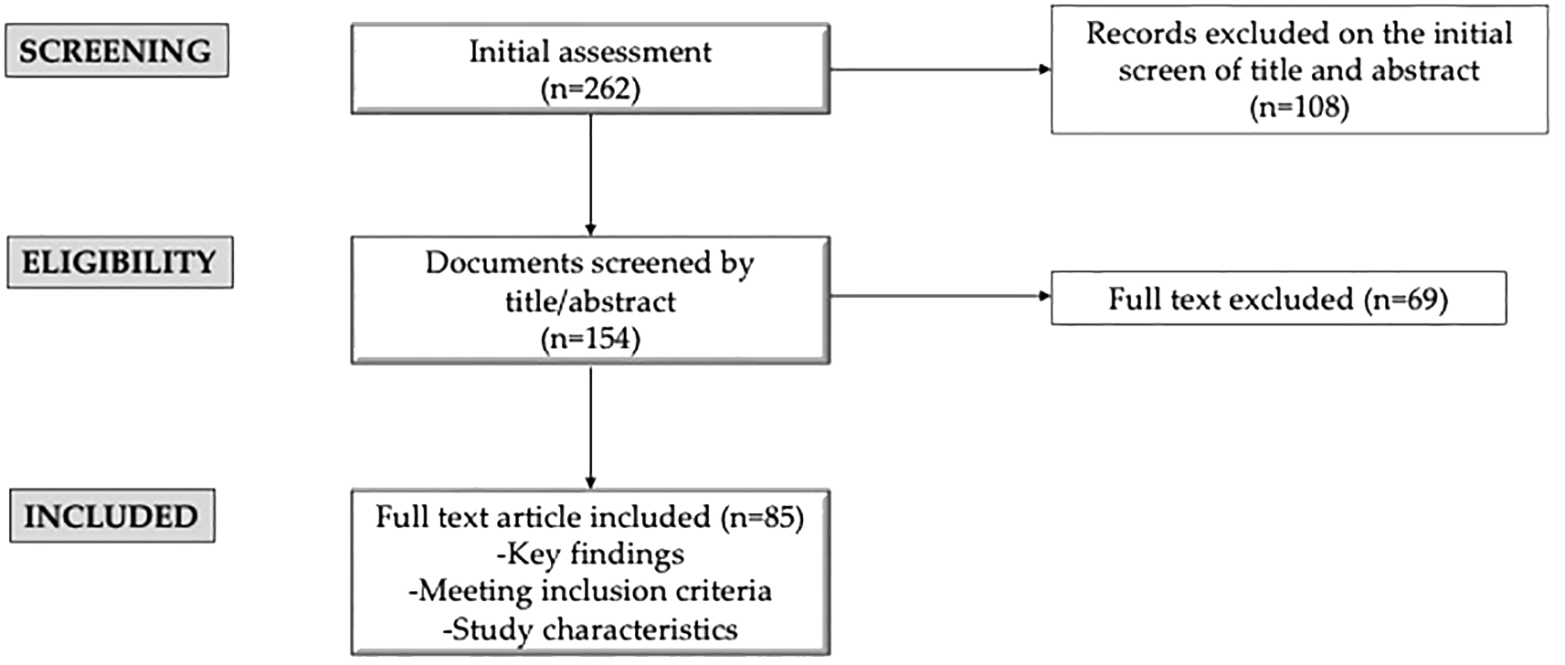
Flowchart of the manuscript selection and exclusion.

Initially, we evaluated 262 articles, refining our selection by screening abstracts (n= 154) and then performing detailed full-text evaluations of relevant studies (n= 85). Each article was rigorously reviewed to allow for a critical analysis. In **Figure 1**, the flowchart of the manuscript selection and exclusion were schematized. 

The draft of manuscript was reviewed by all co-authors, and the final version received approval from everyone.

## 3 Crohn’s disease

CD could affect any part of the gastrointestinal tract from mouth to perianal site, determining a transmural inflammation of the involved area extending from mucosa to serosa. According to the location of the disease, CD can be classified into terminal ileal, colonic, ileocolonic, and upper GI disease (15). The small intestine and the beginning of the colon are the most commonly affected sites: according to literature, terminal ileitis occurs in 71% of pediatric patients and right colitis in another 71% (16). Furthermore, 20% of pediatric patients have a perianal involvement, including skin tags, fissures, fistulas, and/or abscesses (17). According to the different phenotypes of disease, the Montreal Classification categorizes CD as stricturing, penetrating, inflammatory (non-stricturing and non-penetrating), and perianal disease, any of these with a range in severity from mild to severe (18). The Paris classification (19) represents the most recent schema for categorizing IBD in the pediatric population. Notable updates concern the stratification of age at diagnosis, distinguishing between the following categories: A1a for age 0-10, A1b for age 10-17, A2 for age 17-40, and A3 for age >40. As regards the location of disease, it classifies the presence of upper disease, distinguishing between L4a for upper disease proximal to ligament of Treitz, and L4b for upper disease distal to ligament of Treitz and proximal to distal ileum. This system also allows to categorize patients with both stenosing and penetrating disease as B2B3, and identifies any occurrence of growth retardation as G1.

The etiology of CD is multifactorial and not completely known, including several genetic and environmental factors which interact to manifest the disease. Regarding genetics, NOD2, IL23R and ATG16L1 genes have strongly been associated with CD. On the other hand, environmental risk factors implicated with CD include low fiber - high carbohydrate diet, altered microbiome, medications such as non-steroidal anti-inflammatory drugs and smoking (20). In genetically susceptible individuals, the disease onset is triggered by environmental factors that alter the mucosal barrier and the gut microbiota, leading to a loss of tolerance against luminal antigens and enteric commensal bacteria and consequently to an abnormal stimulation of gut immune response (21). This implies a dominant role of the immune system in triggering and maintaining the inflammatory process responsible for a variety of symptoms and complications associated with CD. The aberrant immune response develops not only in bowel but also in extra-intestinal tissues, due to a cross-reaction between epitopes of intestinal bacteria and self-antigens, such as synovium. The most common CD extra-intestinal manifestations in children are arthritis (axial or peripheral), cutaneous alterations (erythema nodosum and pyoderma gangrenosum), eye diseases (episcleritis and uveitis), and hepatobiliary complications (primary sclerosing cholangitis, cholelithiasis and autoimmune hepatitis) (22).

The diagnosis of CD includes a combination of laboratory, radiographic, endoscopic, and pathological findings. When the diagnosis of CD is considered, a complete blood count, a complete metabolic panel, C-reactive protein level, erythrocyte sedimentation rate, and stool studies (i.e., *Clostridium difficile*, parasites and culture) may be useful (15). Fecal calprotectin is a reasonable test to rule out Crohn’s disease for children with equivocal symptoms (sensitivity of 95-100%; specificity of 44-93%). On the contrary, the detection of *anti-Saccharomyces cerevisiae* antibodies (ASCA) in the bloodstream indicates a potential diagnosis of CD. However, their absence does not definitively exclude CD, given their low sensitivity but high specificity in this context. The first-line radiologic investigations are Magnetic Resonance (MR), or Computed Tomography (CT) enterography, or capsule endoscopy. Gold standard in CD activity measurement is the endoscopic study, demonstrating focal, asymmetric, transmural, or granulomatous features.

The assessment of the disease activity is crucial for the management of CD in children, and it is calculated with a standardized score, called PCDAI (Pediatric Crohn’s Disease Activity Index), considering clinical and laboratory features. Treatment of CD in children includes an escalating approach from nutritional interventions to pharmaceuticals (anti-inflammatory agents, immunosuppressive medications, and biologics).

However, in selected cases which present risk factor for poor outcome of disease, biological treatment may be considered as first line therapy (23). This strategy focuses on achieving complete mucosal healing, potentially affecting the natural progression of the disease. Surgical interventions are reserved for treating complications as fistulas, strictures, and abscesses, and can be considered in case of disease refractory to medical treatment (24).

### 3.1 Crohn’s disease and autoimmune thyroid disorders

Although the development of extraintestinal manifestations and autoimmune disorders during CD is well known, the coexistence of autoimmune thyroid diseases and CD has not been well documented, especially in the pediatric population.

The most common autoimmune thyroid diseases in children are Graves’ disease (GD) and Hashimoto thyroiditis (HT). Both of them are characterized by infiltration of the thyroid by T and B lymphocytes, the production of thyroid autoantibodies, and abnormal thyroid function (hyperthyroidism in GD and hypothyroidism in HT) (16).

#### 3.1.1 Correlation between hyperthyroidism/GD and CD

Graves’ disease is characterized by the presence of anti-TSH receptor antibodies (aTSHR), which lead to an excessive production of thyroid hormones, free from TSH control (17). The prevalence of GD in patients with CD ranges between 0.3% - 0.9% (18), but remains unknown in the pediatric population.

A recent bidirectional 2-sample Mendelian randomization study, conducted by Xian et al. (20), investigated the potential causal relationship between GD and IBD distinguishing between CD and UC. The results revealed that genetically predicted CD increases the risk of GD. On the other hand, genetically predicted GD may slightly increase the risk of CD.

A further study, conducted by Szczeblowska et al. (21), examined the levels of antibodies against thyroid-stimulating hormone receptors (aTSHR) in a cohort of 58 patients with documented CD, identifying a significant statistical increase in their level in the studied group compared to the controls.

A literature review, conducted by Shizuma et al. (22), reported three cases of concomitant CD and GD, in which CD diagnosis was prior or simultaneous to GD diagnosis. The authors identified possible common genetic factors between GD and CD, such as PTPN22, CTLA4, and CD40. The role of these non-HLA genes has been extensively investigated in GD patients. Some studies have reported that PTPN22 may influence the risk of developing CD (24, 25), whereas other ones ruled out this hypothesis (26). In a Spanish meta-analysis, the frequency of the minor allele rs1883832T on the CD40 gene was significantly higher in CD patients than in control individuals (27). However, common genetic backgrounds in GD and CD patients may be present, but further research may be necessary.

#### 3.1.2 Correlation between hypothyroidism/HT and CD

Hashimoto’s thyroiditis (HT), characterized by the production of anti-thyroglobulin (TG) and anti-thyroid peroxidase (TPO) antibodies, is the most common autoimmune thyroid disease in children. Both HT and CD are characterized by an overactive immune response and chronic inflammation, suggesting a possible association between their pathogeneses. Data about prevalence of HT in CD patients are variable and not unified and remain uncertain in the pediatric population. Bardella et al. (28) reported a prevalence of 4.4% (4/90); Yakut et al. (29) found a prevalence of 0% (0/33); Cesarini et al. (30) of 2.2% (10/464); Bernstein et al. (31) reported a prevalence of HT in CD patients similar to that of the controls.

Shizuma et al. (22), in their literature review, described ten cases of concomitant HT and CD, in most of which CD diagnosis was prior (5/10) or simultaneous (3/10) to HT diagnosis. One of the reported cases was a young patient with Turner’s syndrome. This condition increases the risk for concomitant HT and IBD (both UC and CD), as shown in a cohort study of 2459 patients (32). Szczeblowska et al. (21) compared levels of antithyroid antibodies aTG or aTPO in CD patients and in a control group, not finding significant differences. Several case-reports have described the co-occurrence of HT and CD (33, 34) but scientific data about their correlation are still lacking, and the association between HT and CD remains under debate.

According to the register-based cohort study, conducted by Räisänen et al. (35), common perinatal risk factors can be found. In particular, preterm birth and postnatal antibiotic use seem to be related to typical pediatric onset of autoimmune diseases, such as autoimmune thyroiditis, but also IBD.

Autoimmune thyroid disorders and CD share an immune-mediated inflammation. The role of tumor necrosis factor alpha (TNF-alpha) is crucial in the CD pathogenesis, but also in thyroid autoimmune diseases. Furtak et al. (36) showed a possible protective role of anti-TNF-α treatment (Infliximab-IFX), a monoclonal antibody used in CD, in the development of thyroid autoimmune disorders. They observed a prevalence of decreased thyroid echogenicity that was ten times higher in CD patients who had not been treated with IFX compared to those who had received IFX. This indicates a significant connection between thyroid disorders and the pathogenesis of CD.

To date, the association between autoimmune thyroiditis and CD can be suggested by their similar features, such as overlapping genetic associations, chronic and usually intermittent inflammation, T cell organ infiltrations, and increasing incidence without specific triggering factors (3, 21, 22, 28, 30). However, further studies are needed to demonstrate their association in childhood.

### 3.2 Crohn’s disease and thyroid cancers

Thyroid cancer (TC) is the most prevalent endocrine malignancy. Papillary thyroid cancer (PTC) accounts for over 80% of all thyroid cancer cases (11, 37). It manifests predominantly in women (with a 3:1 female-to-male ratio) and is more prevalent in Caucasians compared to African Americans. The average age at diagnosis is around 40 years (38). Risk factors for TC include thyroid dysfunction, exposure to radiation at a young age, and genetic predispositions (11). Although TC may occur as an EIC in IBD patients, it is not among the most common in this group. The link between IBD and TC is still unknown, but it is potentially related to systemic inflammation caused by IBD or prolonged immunosuppression from IBD treatments. Moreover, there is a lack of studies focused exclusively on pediatric populations in this context. Our research has gathered data from studies that include IBD patients from adolescence onwards.

Numerous studies have investigated thyroid cancer in the context of IBD. In a European multicenter study, Katsanos et al. (39) observed that over a 15-year follow-up period, the prevalence of cancer among IBD patients was 9.1%, with a majority being EICs. The overall frequency of cancer in this cohort was comparable to that of the general population. Within this context, only two cases of thyroid cancer were identified, both PTC. This study highlighted the increased risk of certain EICs in IBD patients, corroborating findings from other reports (8, 11).

A prospective, multicenter study in Italy by Biancone et al. (5) showed a 5.2% prevalence of thyroid cancer among IBD patients (5). Out of 1209 IBD-diagnosed individuals, 403 developed cancers, with 204 cases in CD patients and 199 in UC patients. Among these cancer cases, dermatological sites were most affected, accounting for 32% of occurrences, while thyroid cancer represented 1.9% of cases. Both perforating CD and extensive UC were identified as significant risk factors for overall cancer and for EIC. This underscores the importance of a nuanced assessment of cancer risk in IBD, considering variables including general population risk factors, disease-specific factors (such as CD phenotype, severity), and the implications of immunomodulator usage.

Additionally, Sonu et al. (38) conducted a retrospective cohort study involving 2686 patients diagnosed with either CD or UC, alongside a control group with inflammatory conditions like diverticulitis and asthma. Over a 21-year follow-up, a statistically significant difference was observed in the age of PTC diagnosis between CD patients, UC patients, and the control groups.

In a population-based cohort study conducted by Taborelli et al. (40) involving individuals aged 18 to 84 years, it was discovered that patients with CD exhibited a heightened risk for certain EICs, specifically TC and non-melanoma skin cancers.

Wadhwa et al. (12) implemented a case-control study, comparing a study group comprising patients with IBD (both CD and UC) against a control group with diverticulitis, to investigate associations with TC. The findings indicate that CD, in contrast to UC, is associated with a heightened risk of thyroid cancer, with age as a protective factor in the development of TC. This suggests the necessity for rigorous monitoring of thyroid disorders in individuals diagnosed with CD.

In the 2018 study by Cao et al. (41), a case-control study and meta-analysis were conducted, involving 1392 adult patients with IBD and a matched control group of 1392 adult patients with diverticulitis. The study entailed tracking health data from a hospital database over a follow-up period of 27,448 person-years, specifically focusing on the incidence of papillary thyroid cancer. It was observed that patients with total IBD or UC had an increased risk of developing thyroid cancer, in contrast to patients with CD, who did not demonstrate this elevated risk.

In the 2017 population-based study by Jung et al. (42), a cohort of patients with IBD (5595 patients with CD) was compared against the general population. The research findings indicated that both male and female patients with CD did not exhibit an increased risk of developing thyroid cancer (TC) compared to the general population.

Yano et al. (43) conducted a study involving 770 patients with CD, both children and adults. Patients were classified by age, sex, disease duration, and age at diagnosis, with CD confirmed through various medical assessments. Findings indicated that overall cancer risk, including TC risk, in CD patients wasn’t higher than in the general population, but there was a notably increased risk for CRC and leukemia.

In **Tables 1**, **2**, researches examining the link between IBD and thyroid disorders and thyroid cancer, respectively.

**Table 1:** Researches examining the link between IBD and thyroid disorders.

First author, year of publication	Study type	Population	Age	Prevalence of thyroid disorders in IBD respect to control group	Methods	Results
Xian, 2023 (20)	Mendelian Randomized study	SNPs associated with GD were obtained from BBJ (212453 individuals). Immunochip data of IBD were extracted from GWAS (2824 cases and 3719 controls).	Adults.	Not applicable	A bidirectional 2-sample Mendelian Randomization has been performed to infer a causal relationship between GD and IBD	Genetically predicted IBD may increase the risk of GD by 24%. Genetically predicted UC may prevent GD
Szczeblowska, 2013 (21)	Observational study	58 patients with CD (>16 y) and 45 patients with abdominal cavity disorders (>16 y).	Adolescents and adults (> 16 years of age).	Prevalence in study group (CD patients): - TSH higher than normal: 7.41% males and 6.45% females. - TSH lower than normal: 3.70% males and 3.23% females. - aTPO positivity: 0% males and 9.68% females. - aTG positivity: 14.81% males and 9.68% females. - aTSHr positivity: 14.81% males and 12.9% females. Prevalence in control group: - TSH higher than normal: 0% males and 1.59% females. - TSH lower than normal: 7.69% males and 1.59% females. - aTPO positivity: 5.13% males and 14.29% females. - aTG positivity: 10.26% males and 14.29% females. - aTSHr positivity: 2.56% males and 3.17% females.	TSH, aTG, aTPO, aTSHR were performed in all the patients in order to evaluate thyroid gland function in patients with CD	The analysis of aTSHR levels suggests a significant statistical increase in their level in the whole research sample
Shizuma, 2016 (22)	Literature review	16 cases of UC and GD, 3 cases of CD and GD, 10 cases of CD and HT, 4 cases of IBD and subacute thyroiditis, 13 cases of IBD and amyloid goiter	16 cases of UC and GD (two cases out of 16 had pediatric age: 17 years old and 14 years old), 3 cases of CD and GD (one case out of three had pediatric age: 14 years old), 10 cases of CD and HT (five cases out of three had pediatric age: 17, 16, 15, 14, and 10 years old), 4 cases of IBD and subacute thyroiditis (none of pediatric age), 12 cases of IBD and amyloid goiter (three cases out of 13: 13, 15, and 16 years old)	Not applicable	Evaluation of prevalence of thyroid dysfunction (hyper- or hypothyroidism), GD, and thyroid cancer between IBD patients and general populations	No obvious differences in the prevalence of thyroid dysfunction, GD, and thyroid cancer between IBD patients and the general population. No correlation between development of thyroid disease and severity of IBD.
Bardella, 2009 (28)	Cross-sectional	90 UC patients, 90 CD patient 117 celiac disease patients	Adults.	Hashimoto’s thyroiditis prevalence in - study group: 2% in UC patients; 4% in CD patients - control group (coeliac disease): 8%	The differences in the distribution of selected characteristics between the three diseases were analyzed using the chi-square test, or parametric or non-parametric analysis of variance	The prevalence of thyroid disease is higher in coeliac patients (8%) but it’s increased also in UC population (2%)
Yakut, 2011 (29)	Retrospective study	146 IBD patients (113 UC and 33 CD patients) vs 66 healthy control subjects	Adults	Thyroid disease prevalence in IBD patients: 9.5% (9.7% in UC and 9% in CD). Thyroid disease prevalence in control group: 1.5%.	Comparison with 2 population (healthy and IBD) using the Fisher’s test and Student’s t-test	4.4% (5/113) of patients with UC has hyperthyroidism vs 0% (0/66) of healthy population 0% has hypothyroidism in UC and healthy population 3.5% (4/113) of patients with UC has thyroiditis vs 1.5% (1/66) of healthy population
Bernstein, 2005 (31)	Retrospective study	3879 UC patients vs 38674 healthy control	Pediatric and adult age, grouped by 0–19 y, 20–39 y, 40–59 y, and ≥60 y	Thyroiditis prevalence in UC group: 1.57% Thyroiditis prevalence in control group: 1.16%; thyroiditis prevalence in CD group: 1.46%; thyroiditis prevalence in control group: 1.29%	Comparison with 2 population (healthy and IBD) using Mantel–Haenszel method	The incidence of thyroiditis in the general population (1.16%) overlaid with the rate of thyroiditis in UC patients (1.5%).
Räisänen, 2021 (35)	Cohort study	11407 children. Of them 245 children (2.1%) received at least one diagnosis of autoimmune disease: 68 (0.60%) with AIT, 19 (0.17%) UC and 10 (0.09%) CD	Pediatric age, with a median of 16.6 years	After a median follow-up of 16.6 years, 2.1% of the studied population were diagnosed with an autoimmune disease, mainly AIT and IBD; with a higher incidence in the age range of 12 to 18 years.	The cohorts of 11407 children born in 2000–2005 were followed-up from birth until 31/12/2018. DM, JIA, and IBD diagnoses in the cohort were obtained using ICD-10; AIT diagnoses were obtained from DPR using ATC	More preterm births among children with autoimmune diseases (8.6% vs. 5.3%, p = 0.035). More use of postnatal antibiotics in preterm children with autoimmune diseases (47.6% vs. 27.7%, p = 0.046)
Furtak, 2020 (36)	Cohort study	61 patients with CD, without known thyroid disorders. Of them, 36 patients were treated with IFX, while 25 patients have never received IFX	Pediatric age	Prevalence of abnormal thyroid patterns in study group (treated group): 16.7% Prevalence of abnormal thyroid patterns in control group (untreated): 44%	An ultrasound examination of the thyroid gland, and levels of TSH, fT3, fT4, aTPO, TRAbs, aTGs were performed in all the patients	10-times higher prevalence of decreased thyroid echogenicity in CD and IFX-naive patients compared to IFX-treated group. A significantly lower TSH levels and higher levels of thyroid antibodies in CD and IFX-naive patients
Casella, 2008 (69)	Retrospective study	162 UC patients vs 5721 healthy control	Adults	Prevalence of thyroid disorders in UC population: 2.5% Prevalence of thyroid disorders in the control group: 7.4%	Comparison with 2 population (healthy and IBD) using chi-square tests	The incidence of thyroid dysfunction in the general population was 7.4% (429 out 5721 subjects), significantly higher than that found in the patients’ population cohort (2.5%)
Snook, 1989 (39)	Retrospective study	858 patients with ulcerative colitis, 378 with Crohn’s disease and 148 with coeliac disease vs 300 healthy control	Adults.	Not applicable	Comparison with 2 population (healthy and affected by gastrointestinal disease) to establish the prevalence of autoimmune disease	4.4% (5/113) of patients with UC has hyperthyroidism vs 0% (0/66) of healthy population

aTG, Antithyroglobulin antibodies, aTPO, Thyroid peroxidase antibodies, aTSHR, Anti-thyroid stimulating hormone receptor antibodies, CD, Crohn’s disease; IBD, Inflammatory bowel diseases; IFX, infliximab; UC, ulcerative cholitis; DM, diabetes mellitus; JIA, Juvenile Idiopathic Arthritis.

**Table 2:** Researches examining the link between IBD and thyroid cancer.

First author, year of publication	Study type	Population	Age	Prevalence of thyroid disorders in IBD respect to control group	Intervention/Methods	Results
Katsanos et al., 2011 (39)	Prospective cohort study	2201 adult patients with IBD, from 20 specific geographical areas across 12 European countries	Adults	Prevalence of EIC in IBD patients (control group): 9.1% Prevalence of thyroid cancer amongst EIC cases(study group): 3.2%.	15-year follow-up	9.1% of patients developed cancer, primarily presenting with a single, extraintestinal neoplasm. Among these, only two instances of TC were recorded, both being cases of PTC
Biancone et al., 2019 (5)	Multicenter prospective nested case–control study	1209 patients with CD or UC, with a diagnosis made at least 6 months prior, regular clinical evaluations, age 17 years or older, no history of previous malignancies	Late adolescents/adults (age 17 years or older)	Prevalence of cancer in IBD patients: 33.3%. Prevalence of thyroid cancer in IBD-related cancer cases: 0.6%	6-year follow-up	Among 1209 patients diagnosed with IBD, 403 developed cancer: 204 of these were CD patients and 199 had UC. In these cancer cases, the DS was predominantly affected, representing 32% of occurrences, while TC constituted 1.9% of the cases.
Sonu et al., 2013 (38)	Retrospective cohort study	Study group: 2686 adult patients diagnosed with either CD or UC and the concurrent diagnosis of thyroid cancer Control group: adult patients with inflammatory conditions (1638 with diverticulitis and 19447 with asthma)	Adults	Prevalence of PTC in UC patients: 0.5% Prevalence of PTC in CD patients: 0.4% Prevalence of PTC in asthma controls: 0.3%	21-year follow-up	There is a statistically significant difference in age of diagnosis of PTC in patients with CD patients diagnosed with PTC at a younger age than those with UC or controls
Taborelli et al., 2020 (40)	Retrospective population-based study cohort	3664 IBD patients aged 18–84 years: 1306 with CD and 2358 with UC	Adults	Prevalence of thyroid cancer in UC patients: 0.04%. Overall cancer prevalence in UC patients (control group): 10.4% Prevalence of thyroid cancer in Cd patients (study group): 0.61%. Overall cancer prevalence in CD patients: 10.8%	Median clinical follow-up for patients with CD: 7.1 years	Specific extra-intestinal cancers, particularly thyroid cancer and non-melanoma skin cancers, were observed to have an elevated risk among patients with CD
Wadhwa et al., 2016 (12)	Case-control study	Study group: adult patients with CD and UC Control group: adult patients with diverticulitis	Adults	Not applicable.	Extracting data from the 2012 National Inpatient Sample database, which provided a stratified 20% sample of discharges from U.S. community hospitals.	CD, as opposed to UC, is linked to an increased risk of thyroid cancer; it is advisable to conduct vigilant surveillance for TC in patients with CD
Cao et al., 2018 (79)	Case-control study and meta-analysis	Study group: 1392 adult patients with IBD Control group: 1392 adult patients with diverticulitis	Adults	Prevalence of PTC in IBD group: 0.79% Prevalence of PTC in patients with diverticulitis (control group): 0.22%	Tracking the health data of 1392 IBD patients from a hospital database, comparing them with a matched control group of patients with diverticulitis, focusing on the development of papillary thyroid cancer over a follow-up period of 27,448 person-years	Patients diagnosed with total IBD or UC exhibited an elevated risk for thyroid cancer, whereas patients with CD did not
Jung et al., 2017 (42)	Population-based study	Study group: 5595 patients with CD and 10049 patients with UC (newly diagnosed from 2011 to 2014) Control group: general population	Adults	Overall cancer prevalence in males with CD (control group): 0.892% Thyroid cancer prevalence in males with CD (study group): 0.026%. Overall cancer prevalence in males with UC (control group): 2.06% Thyroid cancer prevalence in males with UC (study group): 0.504%. Overall cancer prevalence in females with CD (control group): 2.52% Thyroid cancer prevalence in females with CD (study group): 0.455%. Overall cancer prevalence in females with UC (control group): 1.97% Thyroid cancer prevalence in females with UC (study group): 0.455%.	This study utilized the Health Insurance and Review Agency database from the Korean National Health Insurance system, focusing on patients with IBD diagnosed between 2011 and 2014. CD patients - median follow-up time: 2.14 years	Males and females with CD do not have a higher risk of developing thyroid cancer respect to general population
Yano et al., 2013 (43)	Observational population-based study	770 pediatric and adult patients with CD registered in a Japanese hospital between July 1985 and August 2010	Adolescent and adult population	Overall cancer prevalence in IBD patients (control group): 2.47% Thyroid cancer prevalence in IBD patients (study group): 0.13%	Patients have been classified according to sex, age, duration of disease, and age of diagnosis. Diagnosis of CD was confirmed through clinical, endoscopic, radiographic and pathologic evaluations. Observation ranged from the onset of CD to cancer diagnosis, death, or study termination (August 31, 2010).	The overall cancer risk, including thyroid cancer risk, in CD patients was not higher than in the general population, however specific increased risks for colorectal cancer and leukemia were noted.
Biancone et al., 2016 (75)	Multicenter Case Control	IBD patients with a disease history > 6 months, no previous cancer, regular follow-up, age >15 years	Late adolescence and adult population	Study Group (IBD patients who developed cancer): overall cancer prevalence is 33.3%; thyroid cancer prevalence is 5.2% Control Group: 0% prevalence of both overall cancer and thyroid cancer	Each IBD patient was retrospectively crossed matched with two controls	Patients with thyroid neoplasms represent 4,6% of the overall number of ulcerative colitis related cancer cases.
Piovani et al., 2022 (80)	Systematic Review	108 meta-analytic estimates (44 for CD, 38 for UC, and 26 for IBDs)	Adults	Not applicable	Data extraction by two authors with additional consensus. Forest plot for the cancer site association	The study shows a weak association between IBD patients and thyroid cancer
Pedersen et al., 2010 (78)	Meta-analysis of population-based cohort study	Systematic review of eight studies with 17052 patients with IBD available	Adults	Not applicable	Calculation of standardized incidence ratio	Higher incidence of thyroid cancer in IBD, non-specific mention of UC patients

CD, Crohn’s disease; IBD, Inflammatory bowel diseases; UC, ulcerative cholitis; PTC, papillary thyroid cancer; SIR, Standardized Incidence Ratio.

#### 3.2.1 Role of treatments

The primary treatment goal in IBD is the early and sustained healing of intestinal lesions, often involving long-term use of immunomodulators like thiopurines or methotrexate, and TNF-α antagonists. These immunosuppressants can be carcinogenic through DNA alteration, impaired immune control of chronic infections, or reduced immunosurveillance of tumor cells (8). Some immunomodulators, like 5-ASA, have shown chemopreventive properties against colorectal cancer booth in CD and UC patients (44, 45).

On the other hand, thiopurines usage in IBD has been associated with a modestly increased overall cancer risk, but this risk is reversible upon discontinuation of the drug. While in the past, azathioprine usage has been linked to higher rates of lymphoma and non-melanoma skin cancer (46, 47), nowadays, conflicting data exists regarding its chemo preventive effect, with recent studies suggesting a possible benefit (48–50).

Tumor necrosis factor α (TNF-α) antagonists (infliximab, adalimumab, and certolizumab pegol) have demonstrated high efficacy in treating IBD, with all three agents being approved for CD and infliximab and adalimumab also approved for UC. Despite their effectiveness, the potential adverse effects of these agents, particularly the concern regarding treatment-induced immunosuppression potentially elevating cancer risk, necessitate careful consideration. In the past, anti-TNF-α treatments have been associated with increased melanoma rates (51). However, in a comprehensive study by Andersen et al. (51), the relationship between TNF-α antagonist exposure and cancer risk in IBD patients was examined. The study encompassed 3465 IBD patients who had not been exposed to TNF-α antagonists, among whom 6.7% developed cancer, and 81 patients who had been exposed to these antagonists, with 1.8% developing cancer. The initial analysis did not show a significant increase in overall cancer risk associated with TNF-α antagonist exposure. However, upon adjusting for factors such as age, disease duration, and other IBD medications, the risk appeared to increase. Nevertheless, further adjustment for azathioprine use significantly reduced this association. While the study concluded that TNF-α antagonists did not significantly increase cancer risk in this population, it also suggests that any minor increase in overall cancer risk among patients exposed to TNF-α antagonists may be linked to concurrent azathioprine use rather than the antagonists themselves. However, the possibility of an increased malignancy risk over the long term or with accumulating doses of TNF-α antagonists cannot be entirely dismissed, underscoring the need for ongoing monitoring of patients receiving these treatments.

#### 3.2.2 Role of immune system and genetics

Several factors are thought to contribute to the etiopathogenesis of CD and PTC, including genetic, environmental, and immunological elements. In addition to the impact of therapies for CD and the exposure to diagnostic radiation at a young age, Moss et al. (52) suggest that a dietary deficiency in protective micronutrients might be another contributory factor linking CD and TC. CD is characterized by an inappropriate inflammatory reaction, a feature also seen in PTC, particularly through lymphocytic infiltration of thyroid tissue (38, 53). This suggests potential overlapping immunologic pathways linking these diseases. Moreover, specific genetic mutations have been identified in patients with CD, notably in the NOD-2 gene since 2001 (54). Over 30 genetic loci associated with CD have been mapped, playing roles in immune response regulation and epithelial barrier integrity (55). PTC is linked to various genetic mutations, with the most common being RET/PTC1 and RET/PTC3 rearrangements. These are present in a significant portion of adult PTC patients in the United States, Italy, and Canada (38, 56). Additionally, mutations in NTRK1, Ras, and Raf genes have also been recognized (38, 57, 58). Younger PTC patients, particularly children and young adults, exhibit a higher frequency of oncogenic RET and NTRK1 gene rearrangements (59). A study by Sonu et al. (38) highlighted the difficulty in determining the sequential development of PTC and CD due to the small number of patients with both conditions. Further research is needed to better understand the genetic interplay.

## 4 Ulcerative colitis

Ulcerative Colitis is the other main phenotype of IBD. The disease is characterized by diffuse and continuous inflammation of the mucosal layer, and it’s limited to the distal portion of the gastrointestinal tract, from the rectum to a variable proximal portion of the colon. The involvement of the entire colon is called pancolitis which can be a frequent form in pediatric age (60).

The disease course is characterized by remitting and relapsing periods.

In the pediatric population the mean age of diagnosis is around ten years, accounts for 1/3 of the overall IBD diagnosis and affects more males than females with a ratio of approximately 1,5:1 (61).

The exact etiology of UC is still unknown but genetic predisposition and environmental factors play a key role in the pathogenesis of the disease, similarly to what illustrated above for Crohn’s disease. Familiarity is indeed a common feature (61). Other recognized risk factors in the pediatric population are maternal tobacco smoking during pregnancy, passive smoke exposure during early life, as well as altered fibers and sugar intake.

As opposed to Crohn’s disease smoking has protective role for UC influencing the pathogenesis especially in adolescents and young adults (62).

The clinical manifestations are typically divided into gastrointestinal and extra-intestinal. The former are abdominal pain (even nocturnal), tenesmus and bloody diarrhea. The latter involve growth failure, pubertal delay, weight loss, fever, anorexia, and fatigue.

Other common UC extra-intestinal manifestations in children are arthritis (axial or peripheral), cutaneous alterations (pyoderma gangrenosum), eye diseases (episcleritis and uveitis), and hepatobiliary complications (primary sclerosing cholangitis and autoimmune hepatitis). Common complications include digestive system neoplasms (both colonic and biliary due to primary sclerosing cholangitis), osteoporosis and toxic megacolon (1).

Diagnosis is made by clinical manifestation, laboratory findings, imaging (such as intestinal US and enteroMRI), and endoscopic investigation. When UC is considered, we must perform a complete blood count, a complete metabolic panel, C-reactive protein level, and erythrocyte sedimentation rate. Gastrointestinal infections have to be excluded with stool studies (i.e *Clostridium difficile*, parasites and culture) while fecal calprotectin is usually high. Moreover, the presence of pANCA (perinuclear antineutrophil cytoplasmic antibodies) suggests the diagnosis of UC, but their absence cannot rule out the disease (low sensitivity; high specificity). High title of pANCA is associated with more extensive disease (63). The diagnostic confirmation is usually made by histologic data collected from endoscopic investigations.

Recently five diagnostic classes have been identified (PIBD-classes) with the intent of having a more certain diagnosis. Other features such as growth impairment, location, age of diagnosis and behavior of the disease help to have a more specific classification (19).

Current treatment includes medical and surgical therapies, in addition to proper diet and psychological support. Therapy is driven by the severity of the disease, assessed through PUCAI index (Pediatric Ulcerative Colitis Activity Index), and its localization. In mild and moderate forms, it is recommended to use mostly topical or oral 5-ASA after a short course of corticosteroids to induce remission. More severe or non-responsive cases are treated mostly with biologics (19).

Surgery is instead used as a second line therapy especially in poorly responsive subjects. It is usually curative, but the clinician must consider surgery related complications.

Prognosis is generally favorable with good control achieved through medications only; given the relapsing nature of the disease follow-up is mandatory even in case of good clinical response.

### 4.1 Ulcerative colitis and autoimmune thyroiditis

Even for UC the coexistence with autoimmune thyroid diseases is subject of research. From the etiological point of view, UC is not merely an autoimmune condition, but it shares with immune-mediated disease the presence of genetic predisposition, autoantibodies (such as pANCA), and the role of environmental factors (64). The etiologic pathways’ overlap (especially common susceptibility genes and chronic inflammation) between UC and autoimmune disease seems to indicate an increased risk of immune-mediated disorders in UC patients (65–67).

A 2019 report about the adult population by Bar Yehuda has demonstrated how most frequent autoimmune diseases associated with UC are sclerosis cholangitis (1.1% vs 0.7% in CD -OR = 1.55 [1.05–2.31]; p = 0.024), and insulin dependent diabetes mellitus (2.7% vs 1.5% in CD -OR = 1.83 [1.41–2.38]; p < 0.001-). However, thyroiditis does not appear to have higher prevalence among IBD patients than healthy population (1.5% vs 1.2% -OR = 1.67 [0.94–1.44]; p 0.164-) (65).

This lack of correlation between thyropathy and chronic inflammatory bowel diseases has also been confirmed in the pediatric age by a recent Jølving et al.’s article. This review highlights how other autoimmune diseases, such as psoriatic arthritis and spondylarthritis, have higher incidence and earlier onset in the IBD cohort while autoimmune thyropathy seems to have the same incidence as in healthy children (68).

The most common autoimmune thyroid disorders in both healthy and UC children are Graves’ disease, mainly responsible for hyperthyroidism, and HT, principal cause of hypothyroidism.

#### 4.1.1 Correlation between hyperthyroidism/GD and UC

Regarding the prevalence of hyperthyroidism in the UC population, literature is not unanimous. According to Casella et al. there are no differences between UC patients and general population (69).

On the other hand, Snook et al. and Yakut and al. reported a greater risk of hyperthyroidism in UC patients compared with healthy controls (29, 70). Certainly, Graves’ disease and ulcerative colitis share a Th1/Th2 imbalance with increased Th2 activation (71). In Graves’ disease there is increased Th2 activity, resulting in circulating antibodies to TSH-R (18). Increased Th2 activity may explain the antibody production that may contribute to the pathogenesis of UC. However, these mechanisms still do not appear to increase the risk of GD in the UC population. Moreover, from the genetic point of view, UC might prevent GD (OR 0.71, 95% CI 0.58-0.86, p<0.001) (20).

Regarding patients with concomitant GD and UC, there was no evidence of a poorer prognosis (22).

Unfortunately, most data available are from reviews or articles regarding adult population. We don’t have data about the correlation between GD and UC in pediatric patients.

#### 4.1.2 Correlation between hypothyroidism/HT and UC

Even the association between HT and UC does not have an unified correlation in literature. In two different articles (Bardella et al. and Yakut and al.) the risk of HT in UC patients appears to be increased (28, 29). This evidence has not been confirmed by Bernestein et al. and Casella et al. (31, 69).

Noteworthy, some case reports show that the occurrence of HT and UC can be associated with other immune-mediated diseases such as type I diabetes mellitus (DMT1) and Sjogren or DMT1 and autoimmune hepatitis (72, 73). Moreover, patients with Turner syndrome have a greater risk of concomitant HT and IBD (both UC and CD) (32).

As the previous, he correlation between UC and HT has been investigated mainly in adult population. Data for pediatric patients are lacking.

To date, the association between UC and autoimmune thyroiditis appears to be suggested by common etiopathogenetic mechanisms and chronic inflammation. However, particularly in the pediatric population, there is insufficient data to reach a definitive conclusion.

### 4.2 Ulcerative colitis and thyroid cancer

Most of the work we found reported a higher overall risk for patients with UC to develop thyroid cancer in adults (12). One study from Oh et al. reported a higher incidence of thyroid cancer in male patients with UC though the risk for cancer development was similar to the general population (74).

We know that amongst all the neoplasms that patients with UC can develop, thyroid cancer represents a small percentage (75).

Studies concerning thyroid neoplasms as an extraintestinal manifestation of IBD, and more precisely UC in a pediatric population are scarce. The multicenter case-control study from Biancone et al. (75) considered not only adults but also adolescents while describing the association between thyroid cancer and IBD; Piovani and colleagues conducted a review (77) describing a weak association between thyroid cancer and UC in the pediatric population, expressed as a 1.49 RR with a 95% CI. This latter study may be of particular importance since, as stated by the authors, patients referring to a single recruiting center were excluded to avoid bias, including data from observational studies.

#### 4.2.1 Risk factors for cancer in UC

Cao et al. found in their meta-analysis that the risk of thyroid cancer was higher in UC patients than CD ones with a 20-100% increased risk with respect to control population, potentially due to chronic inflammation (78) that may promote an environment capable of promoting cancerogenesis.

Radiation exposure during follow-up may be another causative agent of neoplasms in general, including thyroid cancer, in the general population as described by Piovani et al., but this still has to be verified in a pediatric population (49).

More and more attention is drawn to the role of microbiota in the pathogenesis of immune-mediated diseases, such as UC, and it is known that gut flora alteration may play a role in cancer pathogenesis in these patients (76); to our knowledge it is still be proven whether microbiome alteration plays a certain role in thyroid cancer in the pediatric population (77).

Finally, immunosuppressive drugs may also represent a risk factor (78), as evasion of the immune system is one of the hallmarks of cancer.

The studies documenting the connection between IBD and thyroid disorders, as well as thyroid cancer, are presented in **Tables 1**, **2**, respectively.

## 5 Discussion

IBD and thyroid disorders are two distinct but somehow interconnected conditions that seem to be linked in both adult and pediatric populations.

However, literature data in pediatrics are limited. Specifically, ten different studies have analyzed the relationship between thyroid autoimmune disorders and IBD, yielding varying results. Three studies encompassed childhood, adolescence, and adulthood (21, 22, 31), while two focused on the pediatric population (35, 36). The remaining studies examined only adults. Six of these ten studies (7/10) (20, 21, 28, 29, 36, 70) reported a possible correlation between thyroid disorders and IBD. Two studies (2/10) (21, 36) indicated a significant increase in thyroid disorders in patients with CD compared to the healthy population; three studies (3/10) (28, 29, 70) found an association between thyroid disorders and UC; and one studies (1/10) (20) suggested a potential correlation between thyroid disorders and IBD, emphasizing the role of genetic and perinatal risk factors in the pathogenesis of these conditions. However, three studies (3/10) (22, 31, 69) found no differences in the prevalence of thyroid disorders in IBD patients compared to the general population.

With regard to the relationship between thyroid cancer and IBD, eleven studies have been analyzed. Three studies included both late adolescence and adulthood (5, 43, 75), while the remaining studies focused solely on adults. Five studies (5/11) (12, 40, 78–80) suggested an increased risk of thyroid cancer in patients with IBD, although data are somewhat conflicting. Specifically, one study (1/11) (79) reported an increased risk of thyroid cancer in UC patients; two studies (2/11) (12, 40) suggested a link between thyroid cancer and CD, underscoring the importance of vigilant surveillance for thyroid cancer in CD patients; and one study (1/11) (81) identified a higher incidence of thyroid cancer in IBD, without specifying whether it was associated with UC or CD.

The limited information regarding the relationship between IBD and thyropathies in pediatric patients highlights a significant knowledge gap.

Although the correlation data between thyroid autoimmune disease and IBD are not well established yet, common pathogenic factors have been found.

Genetically, HLA (HLADRB1) and non-HLA genes (JACK/STAT – PTPN22) seem to be involved in IBD and thyroiditis common pathways. CD genetics may increase the risk of GD whereas UC genetics may prevent GD (21).

Moreover, chronic IBD inflammation is associated with a Th1/Th2 imbalance, with a dominance of Th2 responses. This alteration can be found also in immune thyropathies (23).

Environmental factors can also contribute to the onset and the evolutions of both these conditions. In fact, smoking is a major risk factor especially for GD and CD. Even diet associated with chronic inflammation has a valuable role. They can modify the gut microbiota leading to irreversible tissue damage and contributing to a syndrome known as leaky gut. In this condition, toxins leak through the lining of the GI tract and enter the body, contributing to the widespread of inflammation throughout various organs and glands, including thyroid (82).

Furthermore, the nutritional status of individuals with IBD is frequently compromised, with malnutrition manifesting as an imbalance in energy or nutrient intake (83). This includes protein-energy malnutrition, malnutrition related to the disease itself, and deficiencies in micronutrients. While malnutrition occurs in both UC and CD, protein-energy malnutrition and specific nutrient deficiencies are more prevalent in CD due to its ability to affect any section of the digestive tract, particularly the small intestine (84). Chronic progressive inflammation in the intestines can disrupt nutrient absorption, leading to deficiencies in essential vitamins and minerals, such as iodine and selenium. Some of these nutrients are essential for thyroid hormone production and their deficiencies can lead to a lower amount of thyroid hormones available to the cells: iodine, for example, is crucial for thyroxine (T4) and triiodothyronine (T3) production; selenium is a powerful antioxidant, involved in the conversion of thyroxine (T4) in its active form triiodothyronine (T3). Inflammation increases also the production of free radicals, contributing to ongoing oxidative stress, which can promote damage in different areas of the body and contribute to the development of autoimmune disorders like Hashimoto’s (85). Malnutrition can also lead to disturbances in gut microbiome composition, potentially disrupting homeostasis and causing a dysbiotic state. This imbalance may trigger inflammatory responses and serve as an additional contributing factor to thyroid disorders by impairing the absorption of essential elements, such as iodine and selenium (86). Additional factors can contribute to malnutrition in IBD patients, with reduced oral intake playing a significant role in its development. Reduced oral intake may result from various causes, including self-imposed food restrictions, decreased appetite, diminished pleasure from eating, mood changes, and even medical advice, all of which contribute to a worsening nutritional status and, consequently, to nutrient deficiencies and thyroid disorders (87).

Regarding the relationship between thyroid cancer and IBD, some common pathogenic mechanisms can be exploited.

The chronic inflammation typical of IBD, and, on the other hand, the use of immunosuppressive drugs may contribute to the mechanisms of cancerogenesis (78). IBD treatment often involves immunomodulators like thiopurines or methotrexate and TNF-α antagonists, which can increase cancer risk through DNA alterations, impaired immune control, or reduced tumor surveillance. While thiopurines have been associated with a modestly increased overall cancer risk, this risk decreases after discontinuation. TNF-α antagonists are effective in treating IBD but may also elevate cancer risk due to immunosuppression. In addition, different genetic factors involved in immune response regulation have been identified in subjects with thyroid cancer (41, 63–65) and in patients with IBD (61, 62). Specifically, genetic mutations are implicated in both conditions, with mutations in the NOD-2 gene for CD and RET/PTC1 and RET/PTC3 for PTC.

In the end, chronic inflammation and immunosuppressive drugs may contribute to cancerogenesis, however further research is needed to explore these genetic interactions and their role in cancer development. Additionally, the potential impact of radiation exposure on thyroid cancer in pediatric populations remains to be confirmed.

**Figure 2:**
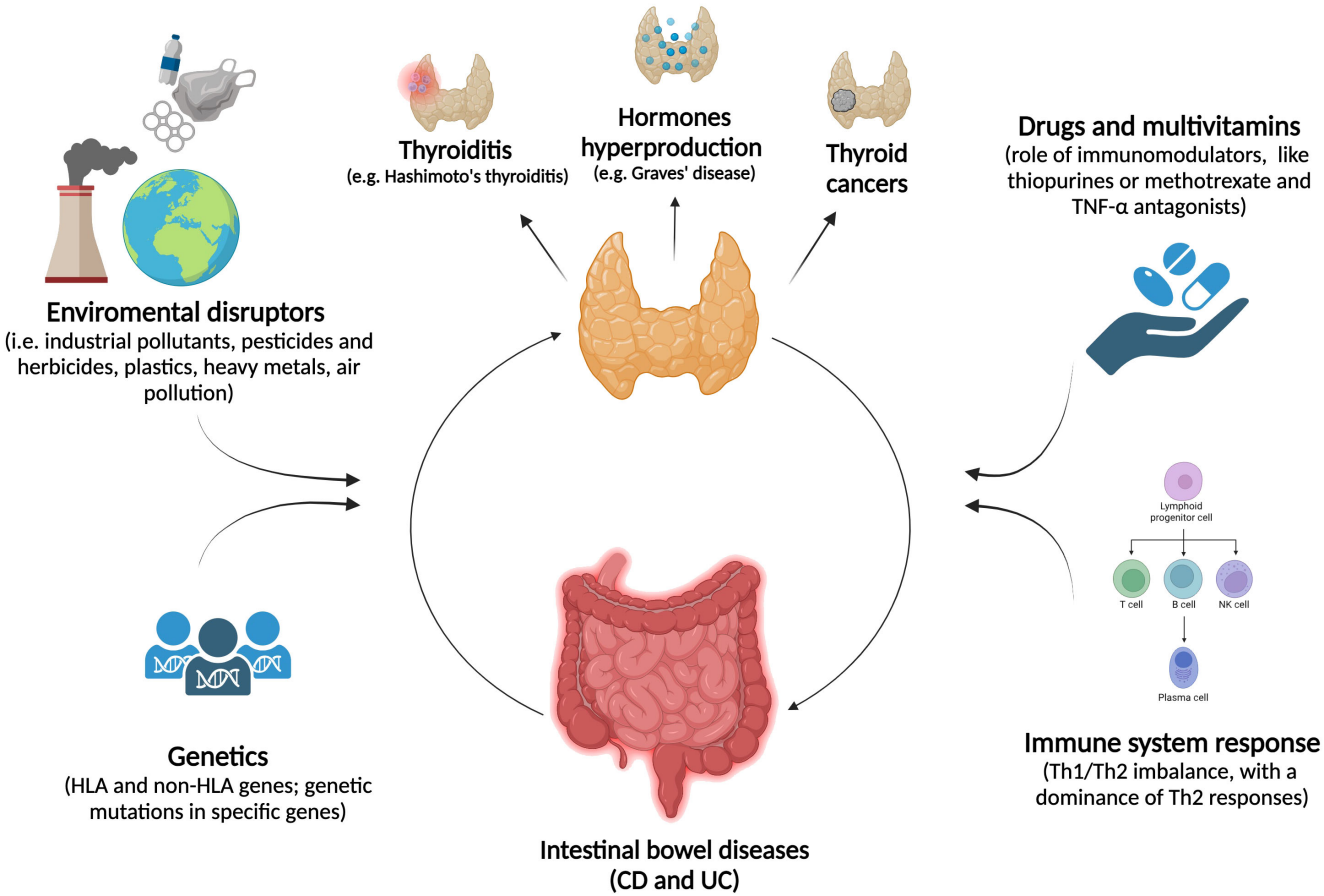
The association between thyropathies and IBD. The relationship between thyroid disorders and IBD is marked by a complex interplay of environmental factors and genetic predispositions, which significantly impact both thyroid function and the digestive system. Immune system disruptions, such as chronic inflammation or imbalances in T-cell responses, combined with the effects of immunomodulatory drugs, may play a key role in the dysregulation of both systems.

In **Figure 2**, the etiopathogenetic similarities between IBD and thyroid disorders are schematized.

We recognized that this review has several limitations. Firstly, we have conducted a narrative review, which, as Gregory et al. (15) pointed out, provides a non-systematic summary and evaluation of existing literature on a specific topic. Due to the non-systematic approach, narrative reviews lack formally established guidelines, which can lead to potential selection biases and typically result in qualitative rather than quantitative syntheses. For example, our review focused exclusively on articles available through PubMed, meaning that studies from other databases or search engines may have been overlooked.

Secondly, discussion on the association between IBD and thyroid disorders is more prevalent in adults compared to pediatrics. On one hand, this allowed our review to include studies on adults, and on the other, it highlighted how the limited number of studies in the pediatric age group could serve as a basis for future research focused on this age group. Physiological differences in hormonal and immunological status in the pediatric age group compared to adults underline the need for personalized monitoring.

Finally, we analyzed the literature on the prevalence of thyroid disorders in IBD. However, to better understand the co-existence of thyroid diseases and IBD, it would also be valuable to examine the IBD prevalence in pediatric patients with thyroid diseases as the first autoimmune disease to appear. Unfortunately, the literature currently available is not sufficient to discuss this specific topic. Further research in this area is essential.

## 6 Conclusions

A significant gap in current knowledge is evident in the literature regarding the relationship between IBD and thyroid disorders. The pathophysiology of the coexistence of these two conditions remains poorly understood, limiting discourse on the topic and underscoring the need for comprehensive, multidisciplinary studies to fully unravel the pathogenic mechanisms underlying this association.

The thyroid plays a critical role during childhood by regulating key developmental and metabolic processes. We recommend incorporating annual thyroid function and autoimmunity tests into the routine follow-up protocol for IBD patients. In addition, a thyroid ultrasound should be considered for patients with positive thyroid antibodies. Regular screening and monitoring of thyroid function are essential to supporting healthy growth in the pediatric population. This approach may aid in the early detection and management of thyroid abnormalities, potentially improving overall outcomes and quality of life for patients. Furthermore, regular follow-up could be instrumental in collecting data and facilitating dedicated studies on this subject.

Future research exploring the genetic and immunologic connections is also essential to enhance our understanding of the interrelation between IBD and thyroid disorders.

## References

[1] Bouhuys M Lexmond WS Van Rheenen PF. Pediatric inflammatory bowel disease. Pediatrics. (2023) 151:e2022058037. doi: 10.1542/peds.2022-058037 36545774

[2] Agrawal M Sabino J Frias-Gomes C Hillenbrand CM Soudant C Axelrad JE. Early life exposures and the risk of inflammatory bowel disease: Systematic review and meta-analyses. EClinicalMedicine. (2021) 36:100884. doi: 10.1016/j.eclinm.2021.100884 34308303 PMC8257976

[3] Wu S Yi J Wu B. Casual associations of thyroid function with inflammatory bowel disease and the mediating role of cytokines. Front Endocrinol (Lausanne). (2024) 15:1376139. doi: 10.3389/fendo.2024.1376139 38872961 PMC11169666

[4] Bandgar TR Shah NS. Thyroid disorders in childhood and adolescence. J Indian Med Assoc. (2006) 104:580–2. 17380823

[5] Biancone L Armuzzi A Scribano ML Castiglione F D’incà R Orlando A. Cancer risk in inflammatory bowel disease: A 6-year prospective multicenter nested case–control IG-IBD study. Inflamm Bowel Dis. (2020) 26(3):450–9. doi: 10.1093/ibd/izz155 31498388

[6] Beaugerie L Itzkowitz SH. Cancers complicating inflammatory bowel disease. N Engl J Med. (2015) 372:1441–52. doi: 10.1056/NEJMra1403718 25853748

[7] Lutgens MWMD van Oijen MGH van der Heijden GJMG Vleggaar FP Siersema PD Oldenburg B. Declining risk of colorectal cancer in inflammatory bowel disease: an updated meta-analysis of population-based cohort studies. Inflammation Bowel Dis. (2013) 19:789–99. doi: 10.1097/MIB.0b013e31828029c0 23448792

[8] Scharl S Barthel C Rossel JB Biedermann L Misselwitz B Schoepfer AM. Malignancies in inflammatory bowel disease: frequency, incidence and risk factors-results from the Swiss IBD cohort study. Am J Gastroenterol. (2019) 114:116–26. doi: 10.1038/s41395-018-0360-9 30333538

[9] Faye AS Holmer AK Axelrad JE. Cancer in inflammatory bowel disease. Gastroenterol Clin North Am. (2022) 51:649–66. doi: 10.1016/j.gtc.2022.05.003 36153115

[10] Zhang H Shi Y Lin C He C Wang S Li Q. Overcoming cancer risk in inflammatory bowel disease: new insights into preventive strategies and pathogenesis mechanisms including interactions of immune cells, cancer signaling pathways, and gut microbiota. Front Immunol. (2023) 14:1338918. doi: 10.3389/fimmu.2023.1338918 38288125 PMC10822953

[11] Mala A Foteinogiannopoulou K Koutroubakis IE. Solid extraintestinal Malignancies in patients with inflammatory bowel disease. World J Gastrointest Oncol. (2021) 13:1956–80. doi: 10.4251/wjgo.v13.i12.1956 PMC8713323 35070035

[12] Wadhwa V Lopez R Shen B. Crohn’s disease is associated with the risk for thyroid cancer. Inflammation Bowel Dis. (2016) 22:2902–6. doi: 10.1097/MIB.0000000000000963 27846192

[13] Khan L. Thyroid disease in children and adolescents. Pediatr Ann. (2021) 50(4):e143–7. 10.3928/19382359-20210322-01 34039175

[14] Gregory AT Denniss AR. An introduction to writing narrative and systematic reviews - tasks, tips and traps for aspiring authors. Heart Lung Circ. (2018) 27:893–8. doi: 10.1016/j.hlc.2018.03.027 29857977

[15] Veauthier B Hornecker JR. Crohn’s disease: diagnosis and management. Am Fam Physician. (2018) 98:661–9. 30485038

[16] Cappa M Bizzarri C Crea F. Autoimmune thyroid diseases in children. J Thyroid Res. (2010) 2011:675703. doi: 10.4061/2011/675703 21209713 PMC3010678

[17] Léger J Oliver I Rodrigue D Lambert AS Coutant R. Graves’ disease in children. Ann Endocrinol. (2018) 79:647–55. doi: 10.1016/j.ando.2018.08.001 30180972

[18] Satsangi J Silverberg MS Vermeire S Colombel JF. The Montreal classification of inflammatory bowel disease: controversies, consensus, and implications. Gut. (2006) 55:749–53. doi: 10.1136/gut.2005.082909 PMC1856208 16698746

[19] Levine A Griffiths A Markowitz J Wilson DC Turner D Russell RK. Pediatric modification of the Montreal classification for inflammatory bowel disease: the Paris classification. Inflammation Bowel Dis. (2011) 17:1314–21. doi: 10.1002/ibd.21493 21560194

[20] Xian W Wu D Liu B Hong S Huo Z Xiao H. Graves disease and inflammatory bowel disease: A bidirectional Mendelian randomization. J Clin Endocrinol Metab. (2023) 108:1075. doi: 10.1210/clinem/dgac683 36459455 PMC10099169

[21] Szczeblowska D Wojtuń S. Thyroid diseases in the course of Crohn’s disease. Gastroenterol Rev. (2013) 2:126–32. doi: 10.5114/pg.2013.34838

[22] Shizuma T. Concomitant thyroid disorders and inflammatory bowel disease: A literature review. BioMed Res Int. (2016) 2016:5187061. doi: 10.1155/2016/5187061 27042663 PMC4794572

[23] van Rheenen PF Aloi M Assa A Bronsky J Escher JC Fagerberg UL. The medical management of paediatric Crohn’s disease: an ECCO-ESPGHAN guideline update. J Crohns Colitis. (2020), jjaa161. doi: 10.1093/ecco-jcc/jjaa161 33026087

[24] Diaz-Gallo LM Espino-Paisán L Fransen K Gómez-García M van Sommeren S Cardeña C. Differential association of two PTPN22 coding variants with Crohn’s disease and ulcerative colitis. Inflammation Bowel Dis. (2011) 17:2287–94. doi: 10.1002/ibd.21630 21287672

[25] Bank S Skytt Andersen P Burisch J Pedersen N Roug S Galsgaard J. Polymorphisms in the inflammatory pathway genes TLR2, TLR4, TLR9, LY96, NFKBIA, NFKB1, TNFA, TNFRSF1A, IL6R, IL10, IL23R, PTPN22, and PPARG are associated with susceptibility of inflammatory bowel disease in a Danish cohort. PloS One. (2014) 9:e98815. doi: 10.1371/journal.pone.0098815 24971461 PMC4074037

[26] Zheng J Ibrahim S Petersen F Yu X. Meta-analysis reveals an association of PTPN22 C1858T with autoimmune diseases, which depends on the localization of the affected tissue. Genes Immun. (2012) 13:641–52. doi: 10.1038/gene.2012.46 23076337

[27] Blanco-Kelly F Matesanz F Alcina A Teruel M Díaz-Gallo LM Gómez-García M. CD40: novel association with Crohn’s disease and replication in multiple sclerosis susceptibility. PloS One. (2010) 5:e11520. doi: 10.1371/journal.pone.0011520 20634952 PMC2902513

[28] Bardella MT Elli L De Matteis S Floriani I Torri V Piodi L. Autoimmune disorders in patients affected by celiac sprue and inflammatory bowel disease. Ann Med. (2009) 41:139–43. doi: 10.1080/07853890802378817 18777226

[29] Yakut M Üstün Y Kabacan G Soykan I. Thyroid disorders in patients with inflammatory bowel diseases. Int J Clin Med. (2011) 02:89–92. doi: 10.4236/ijcm.2011.22018

[30] Cesarini M Angelucci E Rivera M Pica R Paoluzi P Vernia P. Thyroid disorders and inflammatory bowel diseases: retrospective evaluation of 909 patients from an Italian Referral Center. Inflammation Bowel Dis. (2010) 16:186–7. doi: 10.1002/ibd.20964 19462424

[31] Bernstein CN Wajda A Blanchard JF. The clustering of other chronic inflammatory diseases in inflammatory bowel disease: a population-based study. Gastroenterology. (2005) 129:827–36. doi: 10.1053/j.gastro.2005.06.021 16143122

[32] Goldacre MJ Seminog OO. Turner syndrome and autoimmune diseases: record-linkage study. Arch Dis Child. (2014) 99:71–3. doi: 10.1136/archdischild-2013-304617 24064113

[33] Inokuchi T Moriwaki Y Takahashi S Tsutsumi Z Ka T Yamamoto T. Autoimmune thyroid disease (Graves’ Disease and Hashimoto’s thyroiditis) in two patients with Crohn’s disease: case reports and literature review. Intern Med. (2005) 44:303–6. doi: 10.2169/internalmedicine.44.303 15897640

[34] Shah SA Peppercorn MA Pallotta JA. Autoimmune (Hashimoto’s) thyroiditis associated with Crohn’s disease. J Clin Gastroenterol. (1998) 26:117–20. doi: 10.1097/00004836-199803000-00006 9563922

[35] Räisänen L Viljakainen H Sarkkola C Kolho KL. Perinatal risk factors for pediatric onset type 1 diabetes, autoimmune thyroiditis, juvenile idiopathic arthritis, and inflammatory bowel diseases. Eur J Pediatr. (2021) 180:2115–23. doi: 10.1007/s00431-021-03987-3 PMC8195774 33624160

[36] Furtak A Wedrychowicz AM Sladek M Starzyk J. Infliximab therapy could decrease the risk of the development of thyroid disorders in pediatric patients with Crohn’s disease. Front Endocrinol. (2020) 11:558897. doi: 10.3389/fendo.2020.558897 PMC7522276 33042019

[37] National Cancer Institute SEER. Cancer stat facts: thyroid cancer(2024). Available online at: https://seer.cancer.gov/statfacts/html/thyro.html (accessed September 9, 2024).

[38] Sonu IS Blonski W Lin MV Lewis J Aberra F Lichtenstein GR. Papillary thyroid cancer and inflammatory bowel disease: is there a relationship? World J Gastroenterol. (2013) 19:1079–84. doi: 10.3748/wjg.v19.i7.1079 PMC3581995 23467027

[39] Katsanos KH Tatsioni A Pedersen N Shuhaibar M Ramirez VH Politi P. Cancer in inflammatory bowel disease 15 years after diagnosis in a population-based European Collaborative follow-up study. J Crohns Colitis. (2011) 5:430–42. doi: 10.1016/j.crohns.2011.04.013 21939917

[40] Taborelli M Sozzi M Del Zotto S Toffolutti F Montico M Zanier L. Risk of intestinal and extra-intestinal cancers in patients with inflammatory bowel diseases: A population-based cohort study in northeastern Italy. PloS One. (2020) 15:e0235142. doi: 10.1371/journal.pone.0235142 32574216 PMC7310697

[41] Cao J Li H Chen L. Targeting drugs to APJ receptor: the prospect of treatment of hypertension and other cardiovascular diseases. Curr Drug Targets. (2015) 16:148–55. doi: 10.2174/1389450115666141128120053 25438973

[42] Jung YS Han M Park S Kim WH Cheon JH. Cancer risk in the early stages of inflammatory bowel disease in Korean patients: A nationwide population-based study. J Crohns Colitis. (2017) 11:954–62. doi: 10.1093/ecco-jcc/jjx040 28333358

[43] Yano Y Matsui T Hirai F Okado Y Sato Y Tsurumi K. Cancer risk in Japanese Crohn’s disease patients: investigation of the standardized incidence ratio. J Gastroenterol Hepatol. (2013) 28:1300–5. doi: 10.1111/jgh.2013.28.issue-8 23488881

[44] O’Connor A Packey CD Akbari M Moss AC. Mesalamine, but not sulfasalazine, reduces the risk of colorectal neoplasia in patients with inflammatory bowel disease: an agent-specific systematic review and meta-analysis. Inflammation Bowel Dis. (2015) 21:2562–9. doi: 10.1097/MIB.0000000000000540 26296062

[45] Zhao LN Li JY Yu T Chen GC Yuan YH Chen QK. 5-Aminosalicylates reduce the risk of colorectal neoplasia in patients with ulcerative colitis: an updated meta-analysis. PloS One. (2014) 9:e94208. doi: 10.1371/journal.pone.0094208 24710620 PMC3978022

[46] Beaugerie L Brousse N Bouvier AM Colombel JF Lémann M Cosnes J. Lymphoproliferative disorders in patients receiving thiopurines for inflammatory bowel disease: a prospective observational cohort study. Lancet Lond Engl. (2009) 374:1617–25. doi: 10.1016/S0140-6736(09)61302-7 19837455

[47] Ariyaratnam J Subramanian V. Association between thiopurine use and nonmelanoma skin cancers in patients with inflammatory bowel disease: a meta-analysis. Am J Gastroenterol. (2014) 109:163–9. doi: 10.1038/ajg.2013.451 24419479

[48] Rutter MD Saunders BP Wilkinson KH Rumbles S Schofield G Kamm MA. Thirty-year analysis of a colonoscopic surveillance program for neoplasia in ulcerative colitis. Gastroenterology. (2006) 130:1030–8. doi: 10.1053/j.gastro.2005.12.035 16618396

[49] Gordillo J Cabré E Garcia-Planella E Ricart E Ber-Nieto Y Márquez L. Thiopurine therapy reduces the incidence of colorectal neoplasia in patients with ulcerative colitis. Data from the ENEIDA registry. J Crohns Colitis. (2015) 9:1063–70. doi: 10.1093/ecco-jcc/jjv145 26351379

[50] Carrat F Seksik P Colombel JF Peyrin-Biroulet L Beaugerie L CESAME Study Group. The effects of aminosalicylates or thiopurines on the risk of colorectal cancer in inflammatory bowel disease. Aliment Pharmacol Ther. (2017) 45:533–41. doi: 10.1111/apt.13897 27995656

[51] Nyboe Andersen N Pasternak B Basit S Andersson M Svanström H Caspersen S. Association between tumor necrosis factor-α antagonists and risk of cancer in patients with inflammatory bowel disease. JAMA. (2014) 311:2406–13. doi: 10.1001/jama.2014.5613 24938563

[52] Moss AC Brennan AM Cheifetz AS Peppercorn MA. Thyroid cancer and Crohn’s disease: Association or coincidence? Inflammation Bowel Dis. (2006) 12:79–80. 10.1097/01.mib.0000192324.20545.bd 16374265

[53] Kondo T Ezzat S Asa SL. Pathogenetic mechanisms in thyroid follicular-cell neoplasia. Nat Rev Cancer. (2006) 6:292–306. doi: 10.1038/nrc1836 16557281

[54] Ogura Y Bonen DK Inohara N Nicolae DL Chen FF Ramos R. A frameshift mutation in NOD2 associated with susceptibility to Crohn’s disease. Nature. (2001) 411:603–6. doi: 10.1038/35079114 11385577

[55] Van Limbergen J Wilson DC Satsangi J. The genetics of Crohn’s disease. Annu Rev Genomics Hum Genet. (2009) 10:89–116. doi: 10.1146/annurev-genom-082908-150013 19453248

[56] Nikiforov YE. RET/PTC rearrangement in thyroid tumors. Endocr Pathol. (2002) 13:3–16. doi: 10.1385/EP:13:1:03 12114746

[57] Brzeziańska E Pastuszak-Lewandoska D Lewiński A. Rearrangements of NTRK1 oncogene in papillary thyroid carcinoma. Neuro Endocrinol Lett. (2007) 28:221–9. 17627253

[58] Pierotti MA Greco A. Oncogenic rearrangements of the NTRK1/NGF receptor. Cancer Lett. (2006) 232:90–8. doi: 10.1016/j.canlet.2005.07.043 16242838

[59] Bongarzone I Fugazzola L Vigneri P Mariani L Mondellini P Pacini F. Age-related activation of the tyrosine kinase receptor protooncogenes RET and NTRK1 in papillary thyroid carcinoma. J Clin Endocrinol Metab. (1996) 81:2006–9. doi: 10.1210/jcem.81.5.8626874 8626874

[60] Siow VS Bhatt R Mollen KP. Management of acute severe ulcerative colitis in children. Semin Pediatr Surg. (2017) 26:367–72. doi: 10.1053/j.sempedsurg.2017.10.006 29126505

[61] Roberts SE Thorne K Thapar N Broekaert I Benninga MA Dolinsek J. A systematic review and meta-analysis of paediatric inflammatory bowel disease incidence and prevalence across Europe. J Crohns Colitis. (2020) 14:1119–48. doi: 10.1093/ecco-jcc/jjaa037 32115645

[62] Lunney PC Leong RWL. Review article: Ulcerative colitis, smoking and nicotine therapy. Aliment Pharmacol Ther. (2012) 36:997–1008. doi: 10.1111/apt.2012.36.issue-11pt12 23072629

[63] Spencer EA Davis SM Mack DR Boyle BM Griffiths AM LeLeiko NS. Serologic reactivity reflects clinical expression of ulcerative colitis in children. Inflammation Bowel Dis. (2018) 24:1335–43. doi: 10.1093/ibd/izy009 PMC6093192 29718391

[64] Desplat-Jégo S Johanet C Escande A Goetz J Fabien N Olsson N. Update on Anti-Saccharomyces cerevisiae antibodies, anti-nuclear associated anti-neutrophil antibodies and antibodies to exocrine pancreas detected by indirect immunofluorescence as biomarkers in chronic inflammatory bowel diseases: results of a multicenter study. World J Gastroenterol. (2007) 13:2312–8. doi: 10.3748/wjg.v13.i16.2312 PMC4147139 17511029

[65] Bar Yehuda S Axlerod R Toker O Zigman N Goren I Mourad V. The association of inflammatory bowel diseases with autoimmune disorders: A report from the epi-IIRN. J Crohns Colitis. (2019) 13:324–9. doi: 10.1093/ecco-jcc/jjy166 30304371

[66] Kappelman MD Galanko JA Porter CQ Sandler RS. Association of paediatric inflammatory bowel disease with other immune-mediated diseases. Arch Dis Child. (2011) 96:1042–6. doi: 10.1136/archdischild-2011-300633 21903597

[67] Bezzio C Della Corte C Vernero M Di Luna I Manes G Saibeni S. Inflammatory bowel disease and immune-mediated inflammatory diseases: looking at the less frequent associations. Ther Adv Gastroenterol. (2022) 15:17562848221115312. doi: 10.1177/17562848221115312 PMC9340394 35924080

[68] Jølving LR Zegers FD Lund K Wod M Nielsen J Qvist N. Children and adolescents diagnosed with inflammatory bowel disease are at increased risk of developing diseases with a possible autoimmune pathogenesis. Inflammation Bowel Dis. (2025) 31(1):87–94. doi: 10.1093/ibd/izae047 38507606

[69] Casella G De Marco E Antonelli E Daperno M Baldini V Signorini S. The prevalence of hyper- and hypothyroidism in patients with ulcerative colitis. J Crohns Colitis. (2008) 2:327–30. doi: 10.1016/j.crohns.2008.09.001 21172232

[70] Snook JA de Silva HJ Jewell DP. The association of autoimmune disorders with inflammatory bowel disease. Q J Med. (1989) 72:835–40. 2616728

[71] Matsumura K Nakase H Yamamoto S Yoshino T Takeda Y Kasahara K. Modulation of the Th1/Th2 balance by infliximab improves hyperthyroidism associated with a flare-up of ulcerative colitis. Inflammation Bowel Dis. (2009) 15:967–8. doi: 10.1002/ibd.20760 18942759

[72] Najafi M Zamani MM Rezaei N Sabbaghian M. Autoimmunity in inflammatory bowel disease: a case of ulcerative colitis with diabetes mellitus, autoimmune hepatitis and autoimmune hypothyroidism. Turk J Pediatr. (2012) 54:651–3. 23692793

[73] Takahashi K Anno T Matsuda A Kimura Y Kawasaki F Kaku K. Case report: onset of type 1 diabetes mellitus in a patient with ulcerative colitis and Sjogren’s syndrome under euthyroid Hashimoto’s thyroiditis. Front Endocrinol. (2022) 13:836102. doi: 10.3389/fendo.2022.836102 PMC8967944 35370946

[74] Oh EH Kim YJ Kim M Park SH Kim TO Park SH. Risk of Malignancies and chemopreventive effect of statin, metformin, and aspirin in Korean patients with ulcerative colitis: a nationwide population-based study. Intest Res. (2023). doi: 10.5217/ir.2023.00062 PMC12081078 37939723

[75] Biancone L Armuzzi A Scribano ML D’Inca R Castiglione F Papi C. Inflammatory bowel disease phenotype as risk factor for cancer in a prospective multicentre nested case-control IG-IBD study. J Crohns Colitis. (2016) 10:913–24. doi: 10.1093/ecco-jcc/jjw048 26933032

[76] Popov J Caputi V Nandeesha N Rodriguez DA Pai N. Microbiota-immune interactions in ulcerative colitis and colitis associated cancer and emerging microbiota-based therapies. Int J Mol Sci. (2021) 22:11365. doi: 10.3390/ijms222111365 34768795 PMC8584103

[77] Stramazzo I Capriello S Filardo S Centanni M Virili C. Microbiota and thyroid disease: an updated systematic review. Adv Exp Med Biol. (2023) 1370:125–44. 10.1007/5584_2023_770 36971966

[78] Pedersen N Duricova D Elkjaer M Gamborg M Munkholm P Jess T. Risk of extra-intestinal cancer in inflammatory bowel disease: meta-analysis of population-based cohort studies. Am J Gastroenterol. (2010) 105:1480–7. doi: 10.1038/ajg.2009.760 20332773

[79] Cao L. Assessment of thyroid cancer risk in more than 334,000 patients with inflammatory bowel disease: a case-control study and a meta-analysis. World J Surg Oncol. (2018) 16:182. doi: 10.1186/s12957-018-1485-4 30200972 PMC6131907

[80] Piovani D Hassan C Repici A Rimassa L Carlo-Stella C Nikolopoulos GK. Risk of cancer in inflammatory bowel diseases: umbrella review and reanalysis of meta-analyses. Gastroenterology. (2022) 163:671–84. doi: 10.1053/j.gastro.2022.05.038 35643170

[81] Pedersen SE Bateman ED Bousquet J Busse WW Yoxall S Clark TJ. Determinants of response to fluticasone propionate and salmeterol/fluticasone propionate combination in the Gaining Optimal Asthma controL study. J Allergy Clin Immunol. (2007) 120:1036–42. doi: 10.1016/j.jaci.2007.07.016 17935765

[82] Paray BA Albeshr MF Jan AT Rather IA. Leaky gut and autoimmunity: an intricate balance in individuals health and the diseased state. Int J Mol Sci. (2020) 21:9770. doi: 10.3390/ijms21249770 33371435 PMC7767453

[83] Massironi S Viganò C Palermo A Pirola L Mulinacci G Allocca M. Inflammation and malnutrition in inflammatory bowel disease. Lancet Gastroenterol Hepatol. (2023) 8:579–90. doi: 10.1016/S2468-1253(23)00011-0 36933563

[84] Jabłońska B Mrowiec S. Nutritional status and its detection in patients with inflammatory bowel diseases. Nutrients. (2023) 15:1991. doi: 10.3390/nu15081991 37111210 PMC10143611

[85] Jarmakiewicz-Czaja S Ferenc K Filip R. Antioxidants as protection against reactive oxidative stress in inflammatory bowel disease. Metabolites. (2023) 13:573. doi: 10.3390/metabo13040573 37110231 PMC10146410

[86] Fröhlich E Wahl R. Microbiota and thyroid interaction in health and disease. Trends Endocrinol Metab. (2019) 30:479–90. doi: 10.1016/j.tem.2019.05.008 31257166

[87] Duntas LH. Nutrition and thyroid disease. Curr Opin Endocrinol Diabetes Obes. (2023) 30:324–9. doi: 10.1097/MED.0000000000000831 37578378

